# Gel-Based Autologous Chondrocyte Implantation in a Patient with Noncontained Osteochondral Knee Defect at 9-Year Follow-Up

**DOI:** 10.1155/2022/6946860

**Published:** 2022-05-16

**Authors:** Ujjval Deliwala, Sumit Jain Sethia

**Affiliations:** ^1^Deliwala Arthroscopy & Sports Injury Center, Bhavnagar, Gujarat, India; ^2^Department of Orthopedics, Civil Hospital, Bhavnagar, Gujarat, India

## Abstract

Osteochondritis dissecans (OCD) is a disorder of the subchondral bone affecting the adjacent articular cartilage that may lead to cartilage and bone fragment detachment. It commonly occurs in the knee joint, elbow, wrist, and ankle. Although several surgical concepts have been described to treat OCD (fragment fixation, microfracture, autologous chondrocyte implantation (ACI), and mosaicplasty), no gold standard treatment has been accepted for managing OCD. Multiple factors like age, stability of defect, and defect size should be considered while selecting a specific treatment for OCD. Here, we discuss the case of an 18-year-old patient with horizontal and noncontained OCD. The MRI and CT scan evaluations of condylar notch view showed a defect (23 mm × 19 mm × 8 mm) with ICRS grade IV lateral femoral condyle OCD that was successfully managed by gel-based ACI. After 9 years of ACI, the patient was asymptomatic with full range of motions at the knees. Improvement in visual analog scale score, International Knee Documentation Committee score, and Magnetic Resonance Observation of Cartilage Repair Tissue score was also seen at 9 years post-ACI. No further surgical interventions were needed post-ACI.

## 1. Introduction

Osteochondritis dissecans (OCD) is a disorder of the subchondral bone that potentially affects the adjacent articular cartilage leading to detachment of the cartilage and bone fragments [1, 2]. The most common site for OCD is the knee joint, but it also occurs in the elbow, wrist, and ankle [3]. It is presumed that it occurs as a result of repetitive microtrauma, but other factors have also been implicated [4, 5]. The cartilage tissue has limited capacity to repair itself owing to its avascular nature; therefore, if left untreated, it may progress to degenerative osteoarthritis. In patients with a defect that is not fully contained by a rim of healthy cartilage, a specific approach may be required to attach the periosteum and to get a watertight seal in place [6]. Operative treatment is recommended for young patients with unstable lesions or for patients whose lesions have been unresponsive to nonoperative management.

Several surgical concepts have been described to treat cartilage defects, such as fixation of the fragment, microfracture, autologous chondrocyte implantation (ACI), and mosaicplasty [3]. However, no gold standard for optimal operative OCD management has been accepted. The decision to only use a particular treatment depends on multiple factors like age, stability of defect, and defect size. ACI has become one of the most promising surgical techniques that provides repair for hyaline cartilage and can be applied for a larger-sized defect [7].

Here, we report the case of an 18-year-old patient, a student, with clinically symptomatic knee joint, horizontal, and noncontained OCD that was managed by gel-based ACI.

## 2. Case Presentation

An 18-year-old boy experiencing left knee pain and locking instability for 2.5 months was presented at our outdoor department at Arthroscopy & Sports Injury Center, Deliwala Clinic in Kalanala, Bhavnagar. The patient initially received treatment by orthopedic doctors who suggested restriction of activity and medical management followed by knee aspiration three times. Etiology reported was traumatic injury due to a fall from a staircase. The patient was suspected of having anterior cruciate ligament (ACL) with meniscal injury due to the presence of severe pain and locking episodes associated with occasional instability.

**Figure 1:**
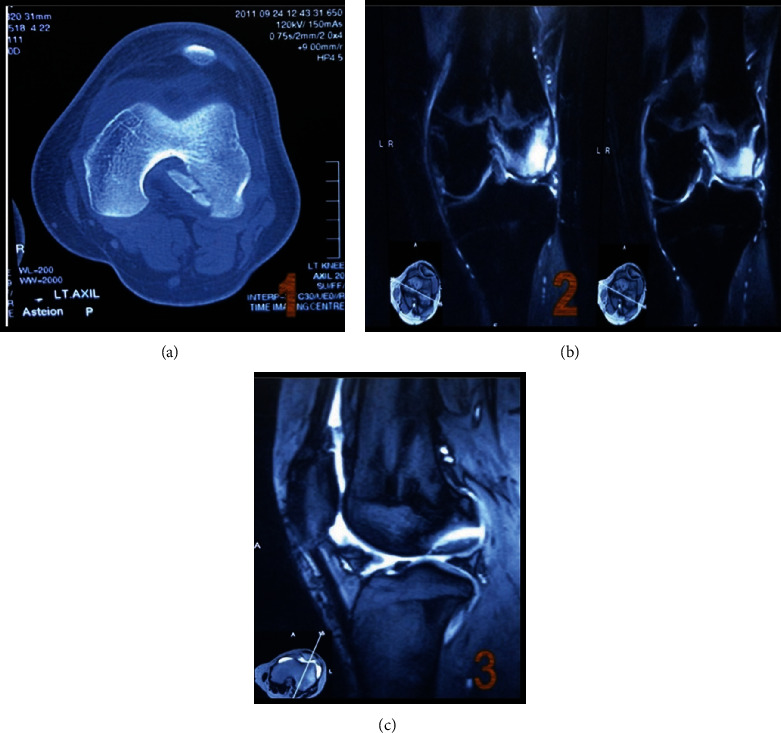
(a) CT scan notch view and (b, c) MRI image showing large defect at the posteromedial aspect of the lateral condyle femur.

Upon clinical examination, we found that the patient had lateral femoral condyle (LFC) tenderness. A positive Lachman test suggested strong evidence of an existing ACL tear. The patient was advised evaluation that was followed by 2 weeks of rest at this stage; however, no physical and clinical improvement was seen after those 2 weeks. An MRI and CT scan evaluation of the condylar notch view showed (Figure 1) a 23 mm × 19 mm × 8 mm defect with ICRS grade IV lateral femoral condyle OCD.

After thoughtful case evaluation and discussion with the patient, we proposed surgical treatment to him as conservative management was no longer effective. After taking informed and written consent, the patient was planned for surgery. We performed ACI in 2011 to treat the defect. The operative therapy consisted of two stages.

Commonly, knee defects are vertical and contained; but in this case, it was a challenge as the defect was horizontal and noncontained. We used autoclaved spoon and X-ray film cut in the required shape and size and curved osteotome to make this big and horizontal defect contained.

In the first stage, we did an arthroscopic assessment and cartilage biopsy on September 29, 2011. Arthroscopy was performed to evaluate the osteochondral defect. Loose bodies secondary to OCD were removed during the procedure. Full-thickness articular cartilage punch biopsy was performed to harvest hexagonal osteochondral cylinders (approximately 6-8 mm in size) with the subchondral bone. All unstable and damaged cartilage was removed with utmost care to avoid penetration into the subchondral bone. Cartilage specimen(s) were then sent to a GMP-certified cell culture laboratory (Regrow Biosciences Pvt. Ltd.) in a sterile container with the culture medium. Harvested cells from the biopsy were then processed for 3-4 weeks in the laboratory to achieve a uniform suspension.

At the laboratory after receiving cartilage biopsy sample, cells were isolated through enzymatic digestion using collagenase solution. Isolation was done in a 25 cm^2^ tissue culture flask containing DMEM (Dulbecco's modified eagle medium) with fetal bovine serum [8]. These isolated cells were seeded and then cultured for 14 days as primary culture. Every 3 days, the medium was changed in the tissue culture flask throughout the culture period. These cells were subcultured repeatedly [8].

Culture process was of 4 weeks when sufficient number of cells were cultured. Approximately, 48 million cells were harvested and transferred into sterile vials. After cell culture was complete, the cells were sent to our hospital maintaining cold chain and sterilization [8].

**Figure 2:**
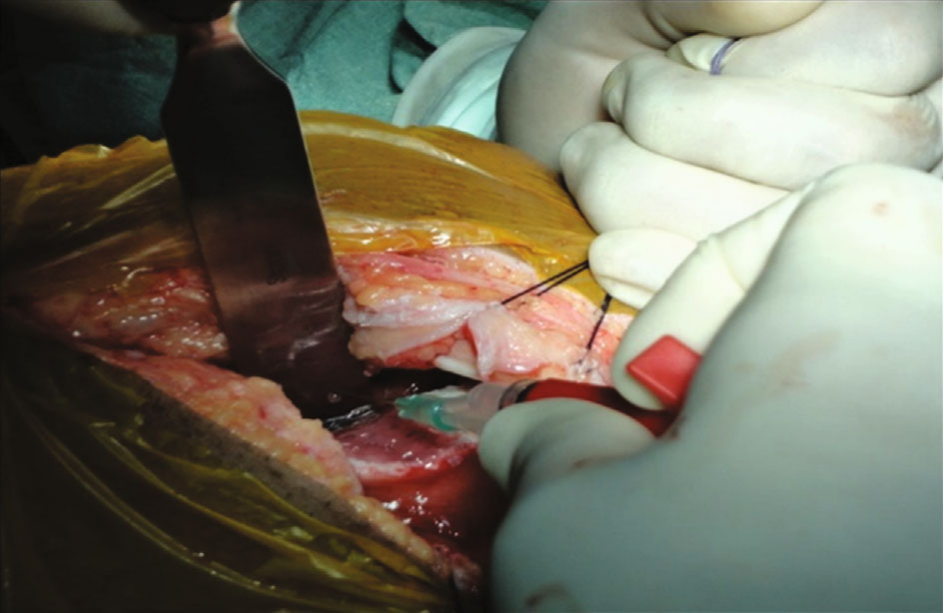
Intraoperative image showing chondrocyte implantation into the defect.

**Figure 3:**
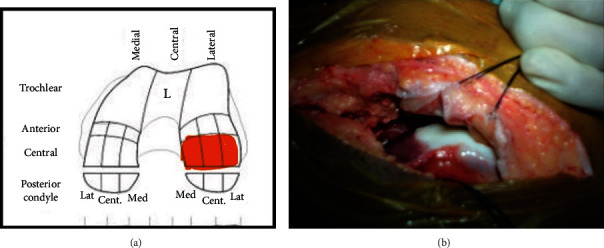
Cartilage mapping showing location of defect: (a) left lateral condyle femur involving central aspect and (b) intraoperative image of defect (ICRS grade IV).

In the second stage, cell implantation (after 4 weeks) was performed with a lateral parapatellar approach on November 01, 2011. We performed debridement of the bone and measured approximate defect size to be 3.5 × 2 cm = 7 cm^2^. The defect was horizontal, in postaspect, and noncontained medially. Arthrotomy was performed, and gel-based ACI (CARTIGROW®) was implanted (Figure 2) directly onto the defect via injection drop by drop while maintaining gravity-eliminating position parallel to the floor to ensure that the implant did not overflow into the surrounding areas (Figure 3). Implant's stability was assessed by moving the knee from full extension to flexion for 10 cycles. Skin and muscle defects were closed in layers, and a compression dressing was applied. The patient followed a postoperative rehabilitation program strictly as given in Table 1 under a trained physiotherapist.

The patient was functionally assessed on visual analog scale (VAS) and International Knee Documentation Committee (IKDC) score preoperatively at 6 months, 1 year, and at 9 years, respectively. Radiological assessment was performed and assessed by Magnetic Resonance Observation of Cartilage Repair Tissue (MOCART) scoring preoperatively and at 9-year post-ACI (Table 2).

**Figure 4:**
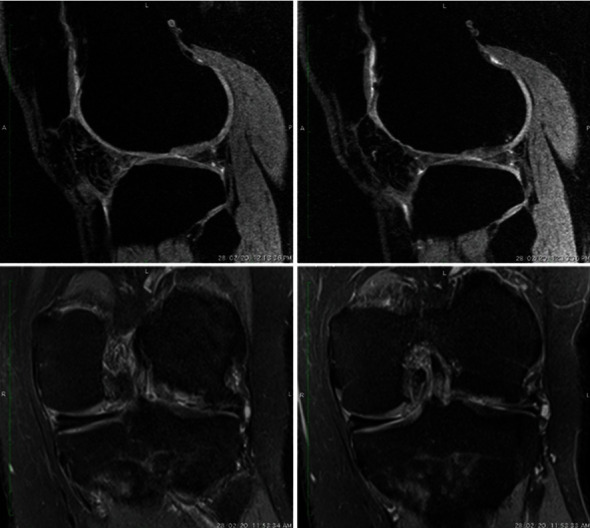
The MRI evaluation posttransplantation showed improvement in cartilage defect.

After 9 years of ACI, he was asymptomatic with a full range of motion (ROM) at the knee. The patient reported no locking episodes or feelings of instability in the knee. After 9 years of transplant, improvement in the VAS and IKDC scores was observed in the patient. The IKDC score improved from 32 to 95, and the VAS score changed from 8 to 0 at 9 years. In addition, the MRI evaluation posttransplantation showed improved cartilage repair (Figure 4). The MRI evaluation showed improved cartilage repair and a MOCART score of 65 at 9 years post-ACI.

## 3. Discussion

This case report shows the 9-year follow-up results with gel-based ACI in an 18-year-old Indian male with lateral femoral condyle OCD with a defect size of 7 cm^2^. A horizontal and noncontained defect is rare; thus, additional skills and management to improve clinical outcomes were required. The patient is doing well with no pain and knee instability 9 years post-ACI. An improvement was found in both VAS and IKDC scores as well as radiological improvement of MOCART scoring in MRI knee at 9-year follow-up.

The goal of treating OCD is to maintain normal function of the knee and delay onset of secondary degenerative complications. Multiple treatment options are available for managing OCD. Conservative treatment for stable grade 1 defects has shown satisfactory results in patients [9–11]; however, surgery may be required in case conservative treatment fails. Arthroscopic surgery with subchondral drilling might be indicated in small lesions, whereas bigger lesions (>2 cm) or multiple loose bodies should be approached with open surgery like microfracture [12, 13]. Microfractures are done in the subchondral bone, which leads to extrusion of marrow elements that stimulate fibrocartilage fill. This fibrous-fibrohyaline tissue is unstructured and lacks biomechanical and viscoelastic features of the hyaline cartilage and has shown short-term improvement of symptoms; but after 2-5 years, this is usually followed by repair tissue failure and gradual deterioration to osteoarthritis and return of symptoms [14]. Moreover, it is deemed to show optimal results only if the lesion's size is <2.5 cm^2^ [15]. Osteochondral autograft transfer system (OATS) is another technique useful for lesions < 2.5 cm^2^ [3]. For larger lesions, ACI is a recommended treatment option [16, 17]. It is a two-stage [16], technically simple solution with no donor site complications for treating full-thickness cartilage lesions of the knee with long-term durability [17].

Peterson et al. reported on an average 13 years of results post-ACI for full-thickness cartilage lesions of the knee [17]. The mean size of the cartilage lesion was 5.3 cm^2^. A total of 74% of patients reported that their clinical status either improved or is stable, and 92% of patients were satisfied and were willing to repeat ACI if required. Presurgical history of meniscal injuries or bone marrow procedures, age at the time of the operation, or the size of the lesion did not affect clinical outcomes in this study.

In this case, we performed gel-based ACI to treat a grade IV defect that resolved clinical symptoms of pain and instability of unlocking knee and improved radiological outcomes at 9 years post-ACI. Gel-based ACI covers irregular defects well owing to its viscous nature. Additionally, a liquid scaffold provides better cell distribution. Several studies reported advantages of ACI over other available treatment options [18–20]. In this case, the arthroscopic evaluation showed a larger defect size (more than 7 cm^2^) over the weight-bearing area. These factors prompted us to use gel-based ACI. As reported, outcome after OD is dependent on the vascular situation and the cartilage surface. The more stable and physiological the cartilage layer, the better the outcome [21]. However, in this case, we operated a horizontal noncontained cartilage defect with sustained clinical improvement even after 9 years post-ACI. Knutsen et al. reported that further surgery could be required in 21% of patients after both ACI and microfracture treatments at 2 years [22]. In this case, the patient did not undergo any surgical procedure 9 years after ACI and is doing well. Good clinical outcomes maintained after 9 years indicated efficacy and durability of the product in this rare lateral femoral condyle defect.

The limitation/current challenges of gel-based ACI technique are that it is expensive and the facility to maintain cell cultures is not easily available. However, each treatment has its limitations, and as of today, no surgical approach has been proven superior; therefore, cartilage defects should be managed in an individualized manner.

## 4. Conclusion

Gel-based ACI provided satisfactory results in terms of both pain relief and knee function rehabilitation for full-thickness large cartilage defect in this patient with OCD that was sustained even at long-term follow-up of 9 years. Thus, for large OCD we recommend two stage Gel based ACI with considering long term good to excellent outcome both clinically and rediologically.

## Figures and Tables

**Table 1:** Rehabilitation protocol of ACI in our center.

Sr. no.	Phases of rehabilitation protocol
1	Phase I (weeks 0-12)
	Weight-bearing (i) Weeks 0-3: non-weight-bearing (ii) Weeks 4-6: partial weight-bearing (30-40 lbs) with progressive use of crutch (iii) Weeks 7-12: progress to full weight-bearing with discontinuation of crutch use
	Bracing (i) Weeks 0-2: hinged knee brace locked in extension—remove for continuous passive motion (CPM) and rehab with PT (ii) Weeks 2-4: gradually open brace at 20° intervals as quad control is obtained
	Range of motion (i) CPM machine functional for 4-6 hours every day for 6 weeks (ii) Set the CPM to 1 cycle per minute—set initially at 0-30° (iii) Increase flexion 5-10° per day until full flexion is achieved (iv) Should be at 90° by week 4 and at 120° by week 6
	Therapeutic exercises (i) Weeks 0-2: straight leg raise/quad sets and hamstring isometrics (ii) Perform exercises in the brace if quad control is inadequate (iii) Weeks 2-6: begin progressive isometric closed chain exercises (iv) At week 6, start weight-shifting activities with operative leg in extension (v) Weeks 6-10: progress bilateral closed chain strengthening and begin open chain knee strengthening (vi) Week 12: begin balance exercises and stationary bike with light resistance
2	Phase II (weeks 12-24)
	Weight-bearing: full weight-bearing with a normal gait pattern
	Range of motion: advance to full/painless ROM
	Therapeutic exercises (i) Advance bilateral and unilateral closed chain exercises with emphasis on concentric/eccentric control (ii) Stationary bike/treadmill/stairmaster/elliptical (iii) Progress balance/proprioception exercises (iv) Start sport cord lateral drills
3	Phase III (months 6-9)
	Weight-bearing: full weight-bearing with a normal gait pattern
	ROM: advance to full/painless ROM
	Therapeutic exercises (i) Advance strength training (ii) Start jogging and sport-specific training at 6 months
4	Phase IV (months 9-18)
	Weight-bearing: full weight-bearing with a normal gait pattern
	ROM: full/painless
	Therapeutic exercises (i) Continue closed chain strengthening exercises and proprioception activities (ii) Sport-specific rehabilitation—running/agility training at 9 months (iii) Return to impact athletics—16 months (if pain-free)

^∗∗^Maintenance program for strength and endurance.

**Table 2:** Functional and radiological outcome scoring.

Duration	VAS score	IKDC score (%)
Preoperative	8	32.18
Postoperative		
At 6 months	3	73.56
At 1 year	0	79.3
At 9 years	0	95.3

## Data Availability

The data used to support the findings of this study are included within the article.
